# Foreign Summary

**Published:** 1839-03-23

**Authors:** 


### FOREIGN SUMMARY.

*Account of a Case of poisoning with Corrosive Sublimate.* By Alexander Wood, Esq.—The patient, a dyer in Musselburgh, named Bell, forty-seven years of age, was admitted, on the 3d of September, into the Royal Infirmary. The following is his case as stated by himself.

On the 26th inst., (nine days before admission,) feeling rather unwell, he applied to an apothecary for a calomel powder, and received from his (the apothecary's) wife about a teaspoonful of a heavy white powder, half of which he took in a glass of whiskey about ten o'clock in the forenoon.

While swallowing it he felt an acute burning sensation in his throat, and immediately after, he was seized with stiffness of the jaws, and vomiting, great pain in the bowels, and the discharge of bloody stools, accompanied with cramps. Ptyalism appeared the same evening; pain in the mouth continued, with ptyalism; and occasionally the pain in the bowels was very severe.

It does not appear that at that time any medicinal means were employed likely to prove effectual in counteracting the effects of the poison. At the time of admission the patient complained more of weakness than of any actual pain, except in the jaws, which were stiff and swollen. The gums were also swollen and spongy. There was considerable ptyalism, and the breath had a strong mercurial fœtor. There was no pain in the bowels, nor was any uneasiness evinced on pressure. He had three stools, according to his own report, of tolerable consistence, and a light colour, within the last twenty-four hours. There was no symptom of any irritation in the urinary passages. The pulse was ninety-six, rather weak, and the countenance denoting exhaustion.

Notwithstanding active treatment, the patient's strength continued to decline. Considerable quantities of fluid blood occasionally escaped from his mouth without any effort like vomiting. This was believed at the time to result from the salivation, rather than from any morbid condition of the alimentary canal. The pulse continued small, and weak, and his whole appearance very much resembled that of a person in the last stage of typhus fever. At no time, however, did he complain of the slightest pain in the bowels. He died on the evening of the 8th inst., (fourteen days after swallowing the powder,) immediately after having passed by stool about six pounds of fluid blood.

In consequence of the circumstances attending the death of this patient, a warrant was issued by the authorities for a judicial examination of the body; and Mr. Watson, surgeon, was directed to perform this duty.

The following were the appearances found on dissection.*

*Mouth.*—The mucous membrane opposite the two last molar teeth of the right side was of a dingy green colour, and in a state of slough. Over one of the tonsils there was a small ulcer. *Pharynx.*—On the surface of the pharynx were two irregular spots, on its posterior aspect, about the size of a shilling, of a brownish colour, and presenting on their surface a number of small elevations.

*Œsophagus.*—About three inches from its termination in the stomach, the œsophagus presented a spot similar to those described as existing in the pharynx.

The *Stomach* contained about six ounces of partly coagulated blood. On its posterior surface, and immediately below the entrance of the œsophagus, a portion of the mucous membrane

* It is proper to state that this report is not the official one, but was drawn up when the body was publicly opened by Mr. Watson, from the appearances then displayed.

was found softened, of a green colour, and part of it hanging loosely in a state of slough. The rest of the mucous membrane of the stomach was of an uniform red colour.

The *Intestines* contained throughout their whole extent, in contact with the lining membrane, a layer of dark-coloured, grumous matter, which was mixed with mucus, and in the duodenum with bile.

The mucous membrane of the duodenum was of a yellow colour, and appeared healthy,—that of the jejunum and ileum was of an uniform red colour. At the termination of the cæcum, and commencement of the colon, round spots existed, less than a sixpence in size, and of a greenish colour, somewhat softened, and irregular on their surface. Near the upper part of the ascending colon a similar spot was observed. On approaching the descending colon, the mucous membrane was observed to be reddened, and irregularly rough on its surface. This appearance increased on proceeding downwards, becoming very marked towards the commencement of the rectum. At some parts blood was seen effused into the submucous cellular tissue, forming little hæmorrhoidal-looking excrescences. Towards the rectum the number of these diminished.

*Chest.*—The large veins contained some coagulated blood. The heart and valves were healthy. The lungs were spongy throughout. On making a section of the posterior part of either lung, a considerable quantity of frothy serum escaped.

*Brain.*—Considerable effusion had taken place into the subarachnoid cellular tissue, stretching the arachnoid membrane across the convolutions of the upper surface of the brain.

The lateral ventricles contained rather more serum than natural. The substance of the brain was of its usual consistence.

*Salivary Apparatus.*—The parotid and submaxillary glands differed but slightly from their natural appearance, seeming only a little softer. The pancreas was natural in appearance and consistence.

*Urinary Apparatus.*—The kidneys contained a slight deposition of yellow granular matter, but no traces of inflammation were observable.

The bladder was quite healthy.

We cannot suppose, that, after the perusal of the above case, any doubt can exist as to corrosive sublimate having been the powder swallowed, so accurately do the symptoms correspond with those detailed by the best toxicologists, as resulting from the action of this powerful mineral salt.

The symptoms of irritation in the œsophagus commenced during the very act of swallowing,—a circumstance which is dwelt upon by Dr. Christison as one, by which poisoning by this substance may be distinguished from that resulting from arsenic.*

This was observed particularly in the case of Mrs. Hodgson, related in a former number of this journal, who, immediately on taking the bolus in which the poison was contained, was attacked with a sense of violent burning in the throat, gullet, and stomach ;* and in a case related by Mr. Blacklock, whose patient "had scarcely got the drug down, when he began to retch, and to complain of constriction in his throat, accompanied with a burning sensation, and difficulty of swallowing." † And in a case recorded by MM. Dumonceau and Planchon, "the patient experienced the caustic effects of the poison the moment he had swallowed it." ‡

The bloody purging and vomiting which occurred during the first few days are also stated by Dr. Christison to follow the action of corrosive sublimate more commonly than that of arsenic, "obviously," he adds, "because the former is a more powerful irritant."

But, independently of every other proof, the profuse salivation under which the unhappy patient laboured on his admission into the hospital, and the peculiar fœtor of the breath, leave no doubt that he was under the influence of some compound of mercury; while the immediate appearance of the irritant action, along with the other symptoms detailed, lead to the conclusion that corrosive sublimate was the active preparation employed.

The chief peculiarities of the case, in a medical point of view, are, *first*, the early period at which salivation commenced.

It is doubted by Dr. Christison whether salivation has ever occurred sooner than the beginning of the second day ; and it is suggested, that, in a case described by Mr. Anderson of Belfast, where it was supposed to have taken place only nineteen hours after the poison had been taken, the salivation arising from inability to swallow, owing to soreness of the throat, had been mistaken for the true mercurial ptyalism. §

In the case of Bell, however, although we have only the statement of the patient himself, that salivation occurred at tea-time, (that is seven hours after,) on the day on which the poison was swallowed, yet, after repeated interrogations, I felt convinced that in this account he was correct. That, in his case, at least, there was no difficulty in swallowing, is proved by his having drunk diluents copiously, *and without any difficulty*, during the earlier stage of the disease.

The length of time during which the patient survived, while the disease was proceeding to its fatal termination, is a rare occurrence.

With regard to the ordinary duration of the disease when the patient dies of the primary symptoms, Dr. Christison observes, that "it varies from twenty-four to thirty-six hours." "It is probable," he continues, "that a few may last three days ; but only one instance has come under my notice where the duration was greater ; and in that instance, which is described by Dr. Venables, life was prolonged under great agony from

* Christison on Poisons, p. 35.

* Edin. Med. Journal, xxxvi. p. 92.

† Vol. xxii. 438.

‡ Journal de Medecine, xlix. p. 36.

§ Op. cit. p. 363.

pain in the belly, bloody vomiting, diarrhœa, and suppression of urine, but without salivation for eight days."*

It is true, that, in the case of Bell, the disease could not strictly be said to be in the first stage, salivation having already commenced; but we think the symptoms during life, as well as the appearances found after death, satisfactorily show that the patient died, not from any of the secondary effects of the poison, but from the consequence of the primary irritation, caused in the stomach by its use.

In the case of Bell there were no local symptoms at the time of his admission into the hospital, or during his stay there, which could have led us to suspect the existence of the serious organic lesion which the *post mortem* examination disclosed, and, in this respect, it differed widely from the case related by Dr. Venables. Though the bowels were inclined to be open, yet diarrhœa could not be said to exist, and at no time did the patient ever complain of the slightest pain in the bowels, or betray any uneasiness when pressure was made upon the abdomen. This I consider a fact of great practical importance, from its illustrating the extent to which disorganization of the intestines may proceed without the occurrence of any *local* symptoms to direct our attention to the lesion, provided always the *mucous membrane alone* be implicated.

By Dr. Stokes† it is stated, that "in acute gastritis," (as the result of swallowing acrid poison,) "there is generally great tenderness of the epigastrium, so that the slightest touch, the weight of the bed-clothes, or any muscular effort, will produce severe distress. Anxious to ascertain on what foundation this opinion rested, I have examined, as far as was in my power, the isolated cases recorded in the various medical periodicals, and I nowhere find that stress laid on these symptoms, which might naturally be expected, did they usually appear as prominently as the statement of Dr. Stokes would lead us to believe. This is particularly taken notice of by Dr. Mackintosh,‡ who illustrates the frequent absence of all local symptoms, where, nevertheless, serious organic lesion may be going on, by the case of a soldier of the 17th foot, who died eight or ten days after swallowing two drachms of this very substance, and in whom the stomach was found in a state of ulceration.§

That ulceration of the stomach frequently does exist without any local symptoms to direct us to it, few, we think will be disposed to deny.

In Dr. Abercrombie's work on the stomach, several cases of ulceration, proving fatal by perforation, are related, where the patients had suffered only from slight and occasional dyspepsia. And the same author quotes a remarkable case, occurring in the practice of Dr. Kellie, of Leith, where sudden perforation of the stomach took place, in a "strong and healthy-looking servant girl, without any previous complaint whatever." The stomach in this case exhibited extensive disease, which must have existed for a considerable period antecedently to its fatal termination.

In a well-marked case of gastritis, which lately came under my notice, I directed my attention particularly to the effect of pressure applied to the abdomen, but failed to observe that extreme sensibility which has been described. The patient had attempted to poison herself with a large quantity of laudanum, and after evacuation of the contents of the stomach, strong stimulants, chiefly, I believe, the *aqua ammoniæ*, in large and repeated doses, had been exhibited. On the third day symptoms unequivocally pathognomic of *gastritis* appeared; but, although the general sensibility was completely restored, the patient suffered very slightly, if at all, from pressure on the abdomen. As the disease yielded to free depletion, there was no opportunity of verifying the diagnosis by actual examination; but the symptoms were such as to convince numerous practitioners who saw the case, that it was one of acute gastritis. I have dwelt thus particularly on this circumstance, because I am inclined to think that pain on pressure is very frequently absent, even in acute cases of inflammation of the gastro-intestinal mucous membrane, and, indeed, that it is rarely observed unless when the serous membrane is also implicated.*

In most of the cases which have been recorded, where any quantity of corrosive sublimate has been swallowed, the patients suffered much from irritation of the urinary passages; and to such a degree does this at times proceed, that even the external parts are found to be black and swollen. In the case of Bell, however, nothing of this kind was complained of, and the bladder after death presented none of those deviations from the natural appearance which, in similar cases, have generally been observed.

In the necroscopic appearances there is perhaps nothing more than might have been expected; but I cannot avoid drawing attention to the spots which were found at the termination of the cæcum, and commencement of the colon. They are described in the report given of the dissection, as about the size of a sixpence, of a greenish colour, somewhat softened, and irregular on their surface.

Though varying somewhat in appearance from those described as existing in the pharynx and œsophagus, yet their nature would seem to have been identical, and very similar to the one observed by Dr. Venables, which, in his case, existed on the superior surface of the stomach, about

* Op. cit. p. 362.

† Cyclopædia of Practical Medicine, Art. Gastritis.

‡ Practice of Physic, Vol. i. p. 297.

§ By the kindness of Dr. Thomas Wood, I have been permitted to examine a preparation of part of the intestines of this patient, along with several drawings illustrative of the case. The large intestines were covered with numerous ulcers, similar to those described as existing in the acute dysentery of tropical climates, and a great part of the mucous membrane was hanging loose, in shreds, apparently in a state of slough.

* In further illustration of this, see cases recorded in Vol. xliv. of this Journal, and Craigie's Practice of Physic, Vol. i. p. 888.

midway between the cardiac and pyloric orifices, and which that gentleman describes, as "a very remarkable kind of opaque, yellowish white spot, of an irregular form, and about the size of a sixpence." I am inclined to believe that these spots resulted from softening of the intestinal mucous membrane, and that the appearances described as existing in the descending colon and rectum were produced in the same way rather than by the effusion of lymph.* "The effusion of lymph as a result either of natural inflammation, or of that caused by poison," is stated by Dr. Christison to be rare; "at least, by no means so common as would be supposed from what is said in systematic works."†

In a medico-legal point of view, this case, though one of considerable interest, is easily disposed of. The length of time which had elapsed between the swallowing of the poison, and the death of the patient, afforded little hope of its being detected in any part of the body, and this, as far as I know, was not attempted. The question has been suggested, how far a medical man is entitled to say, that death, in any case, has resulted from the effect of poison, unless he can actually detect its presence in the body?—But, with every wish to submit to the authority of those who have maintained this opinion, there are, we cannot but think, cases where, independently altogether of the moral evidence, the witness can affirm, that the symptoms preceding, and the appearances detected after death, have resulted from the action of some powerful poison, and even, as might I think in this case have been done, to speak decidedly as to what poison was.

The statement made by the deceased, on his admission into the hospital, conveyed the impression that the powder which caused his death was given him by mistake for calomel. I believe it turned out, on inquiry, that he had sent expressly for corrosive sublimate, having been informed by a fellow-workman that it was an excellent cure for a form of venereal disease under which he laboured.

This confirms an opinion expressed at the time of the *post mortem* examination, that the existence of this disease should have been mentioned in the official report drawn up, as it was extremely likely, that, had a trial taken place, this would not have proved an unimportant circumstance, especially when death was caused by a preparation of mercury.

It is impossible to be too minute in noticing the *leading features*, at least of *every* alteration from the healthy state, which may be found in bodies, on which we are required to furnish a legal report; and in support of this opinion, the authority of Mr. Watson himself may be quoted, who, in reporting two cases in this Journal, where he was directed by the authorities to examine the bodies, premises, "that the task is often difficult, and requires the utmost attention, even to very minute particulars; for circumstances apparently trifling in the medical evidence, often turn out to be of great importance in elucidating and connecting the moral evidence, either of the innocence or guilt of the accused."*

I cannot conclude this case without remarking, that the fact of the deceased having sent a written line to the apothecary for the drug, by no means frees that individual from blame, as it certainly would have been only a necessary precaution, before dispensing such a deadly poison, to have inquired as to its ultimate destination. Cases of poisoning from the carelessness of apothecaries are, I understand, becoming alarmingly frequent. Within a very short time two have occurred in my own experience, where, from the want of a qualified assistant, in the absence of the apothecary himself, a wrong medicine was dispensed, which in one case proved fatal, and in the other gave rise to severe and protracted suffering.

In the establishment where Bell procured the poison, the individuals in charge were the wife of the apothecary and her son, a linen draper's apprentice.—*Ed. Med. and Surg. Jour.*

---

*On Simple Chronic Ulcer of the Stomach.* By M. Cruveilhier.—The researches of M. Cruveilhier on the pathological anatomy of the stomach, lead him to conclude that there exists occasionally simple chronic ulceration of that organ, essentially different from cancerous ulceration of the stomach, but often presenting similar symptoms. There is, indeed, no pathognomic symptom, which will enable us generally to distinguish the two diseases with precision, although there are circumstances which will guide our diagnosis. The natural termination of the two diseases differs essentially; as, whilst cancer of the stomach has an inevitably fatal tendency, simple chronic ulcer will often heal under a soothing treatment, and after a careful abstraction of all irritants. Yet this disease will sometimes terminate fatally by causing perforation of the stomach, or excessive hæmorrhage; and even when the ulcer has healed, it will leave permanent ill consequences in some cases, producing a contraction of the stomach unfavourable to the passage of food, or attacking the coats of the arterial trunks which lie beneath the cicatrix.

1. Simple chronic ulcer of the stomach consists in a spontaneous loss of substance, generally circular, with sharp borders, dense and gray at the bottom, and of variable dimensions. There is rarely more than one, and this is situated in the small curvature or posterior part of the stomach. When it attacks the pylorus, it assumes

* This peculiar softening of the mucous membrane, I think, I have occasionally observed, though in a slighter degree, on examining the intestinal mucous membrane of children who died of some disease, for the cure of which calomel had been very freely administered, and I have more than once heard *that* termed effusion of lymph which I suspect would have shown itself, on closer examination, to have been softening of the mucous membrane.

† Op. cit. p. 122.

* Vol. xxxvi. p. 86.

the form of a zone. Its progress is slow, and as it extends in surface it increases in depth.

2. This kind of ulceration presents the same characters as cutaneous ulcers produced by a constitutional or local cause. It frequently resembles a syphilitic ulcer, but there is no ground for attributing to it a syphilitic origin.

3. Simple ulcer of the stomach may be distinguished from cancer by the absence of the hypertrophied and hardened base which accompanies scirrhous ulceration.

4. All the causes of gastritis are capable of producing simple ulcer of the stomach; but it is not uncommon to find this lesion in the bodies of persons who, during life, presented no symptom of it whatever. More generally, however, symptoms similar to those of scirrhus characterize it. Thus there is a failing or capricious appetite, insurmountable lowness of spirits, pain at the epigastrium, increased during digestion. This pain frequently extends to the corresponding portion of the vertebral column, where it is felt with greater intensity than anteriorly. Emaciation, constipation, nausea, and vomiting of food, as well as vomiting of blood or a black matter, present a train of symptoms so similar to those of cancer, that it is only the experience of the effects of remedies which enables us to pronounce on the exact nature of the disease. It may, perhaps, be said, that in cases of simple ulcer, the patient is not so completely weighed down by the symptoms as in scirrhus.

5. If we examine the surface of the ulcer under water by the aid of a good magnifier, or even with the naked eye, we see a number of vascular orifices, some obliterated, others still open. It is from these orifices that the blood is poured out, which sometimes produces alarming hæmatemesis. The black, sooty colour of the vomited matter arises from blood which has remained some time in the stomach, and has undergone the action of the gastric juice. When the ulceration attacks a vessel of considerable size, a quantity of blood may be poured into the stomach and bowels, the loss of which is sufficient to cause death. This termination is more frequent in simple ulcer than in cancer. Sometimes, indeed, the ulcer is completely cicatrized in every point except that which corresponds to a perforated vessel. In this case, the giving way of the coagulum may produce a fatal hæmorrhage.

6. An absolute diagnosis between this disease and cancer is of less consequence, because the treatment should be nearly the same. Repose of the stomach, as complete as the supply of the absolute wants of nature will allow, is essentially necessary. It is impossible to say beforehand what diet the ulcerated organ will bear; this must be determined in each case by careful experiment. Some will bear fish or white meat; others, veal or chicken-broth; whilst others will endure merely water containing sugar or gum in solution, or even simple water. The treatment should be commenced by enjoining on the patient complete abstinence even from liquids for twenty-four hours. If there is pain at the epigastrium, leeches should be applied, and should ℥ followed by a bath. The day after, a milk diet should be tried. A few spoonsful of new milk taken occasionally will often suffice, and agree well with the patient; if this is not the case, recourse must be had to gelatinous or farinaceous food, and the desires of the patient must be consulted. His natural instinct will often guide us to the discovery of the most proper aliment. Calcined magnesia is occasionally useful; opium rarely: sugar is in general injurious. Baths of gelatine are a very powerful auxiliary, and are much more useful, when they are continued during a space of two, three, or four hours. In this, as in many other chronic affections, it is important not to prolong the system of abstinence too far. Occasionally a change to more stimulating food, as game, will be of service. It is in the details of such a case that there is most room for the exercise of the sagacity of the practitioner. Quality and quantity of aliment; number and period of meals; temperature; period of exercise; excretions: all these points require the minute attention of the physician, and it is by attending to them, rather than by the action of medicines, that we may hope for the best results.

The cicatrices of the ulcers are all fibrous, very resistent, and consequently fragile. It is erroneous to state, that the losses of substance of mucous membranes are replaced by an accidental mucous tissue: a fibrous tissue not covered with a mucous layer replaces the portion of destroyed stomach. Recovery from ulceration, by no means renders the patient less exposed to perforation and hæmorrhage.

The cicatrization of losses of substance of the stomach, similar to the same process occurring in the skin, is performed in two ways: 1, by the drawing together of the edge of the wound; 2, by the production of a fibrous tissue. Inconsiderable losses of substance are cured exclusively by the first method, and then the cicatrix of the stomach is represented by a linear stroke or by a small depression, with circular puckering and radiated folds. More considerable losses of substance leave a circular depression, as if made by a punch, which has a fibrous bottom, limited by a border of mucous membrane more or less projecting.

Perforations may happen during the period of improvement of the ulceration, when the necessary adhesions are not established, and then it may be the result, 1st, of a new ulceration, which may occur on the very bottom of the cicatrization, or upon a point of its circumference; 2dly, from a default of extensibility, from the fragility of the cicatrix, which is broken in consequence of distension of the stomach, either by gas or by aliment, or from violent vomiting.

Hæmorrhage may take place: 1st, during the period of increase of the ulcer; it is then the result of erosion of the arterial walls; 2dly, after the cicatrization, and then sometimes it is produced by the fall of an obstructing clot; sometimes it is in consequence of an ulceration which invades a portion of the cicatrix, or a process of erosion limited to a vessel.

Death is the certain and inevitable consequence of perforation. The hæmorrhage may be either rapidly fatal, or the patient may sink from a succession of attacks. The blood is generally passed by vomiting; it is often passed both by vomiting and by stools. In some cases altogether by the stools which are like ink: in these cases it is probable that the blood is furnished in small quantities, and is in some measure digested.

Those patients who have been cured of this disease are prone to a return from the slightest causes. M. Cruveilhier has seen it reproduced in the same patient three times, at intervals of from two to four years. The knowledge of a patient having recovered from such an attack, should indicate great caution in the employment of irritating medicines. Two cases are given: we select the last, because it clearly points out the importance of bearing in mind this important observation.

A female, aged sixty, entered the hospital on the 23d of September, 1834, for a prolapsus of the rectum. The attention being directed to this point only, the remedies for such an affection only were ordered. Some days after, upon the patient complaining of a bad taste and of habitual constipation, she was ordered twenty-four grains of ipecacuanha, with relief. On the following day, she complained that her food produced a very painful sensation in the epigastric region, and it was at this period she gave the history of her case. She had been seized eighteen months before with vomiting, pains in the stomach, and fever. The vomiting was produced by the ingestion of food, and occurred from time to time, accompanied with epigastric pains; these were so violent as to give the sensation of an animal in the stomach. Pressure upon the epigastric region caused a very acute pain, but no tumour could be discovered. Cataplasms and milk diet were ordered. On the 4th of October, she was suddenly seized with an acute abdominal pain, agitation, and anguish. The following morning there were a distressed and discoloured countenance, miserable pulse, cold perspiration, acute sensibility of the abdomen; more especially of the epigastrium and the left hypochondrium, nausea and hiccup. M. C. diagnosticated a perforation of the stomach: fifteen leeches and cataplasms were applied, but the patient died during the night.

*Dissection.* The intestines were injected and covered with a yellow, false membrane, mixed with bile and alimentary matter. The pelvis contained several ounces of a turbid serum, in which were several seeds and skins of grapes. The intestines were united together. The stomach presented in front, very near the great curvature, not far from the pylorus, a round perforation, about two lines in diameter. The stomach when opened presented, on a level with this perforation, a recent ulceration of an oval form. Independently of this recent ulceration, there existed upon the posterior wall, on a level with the pancreas, a cicatrix formed by this organ, but covered by a layer of smooth fibrous tissue. The mucous membrane formed a circular pad, but it was in a manner continuous with the tissue of the cicatrix. M. C. believes that the irritating nature of the emetic caused the return of the disease.

Many other cases are related, illustrating the termination of this disease. Four cases are detailed in which the stomach was perforated by the ulceration; its contents escaped into the cavity of the abdomen, and death ensued in a few hours. Two cases are given in which the disease terminated by fatal hæmatemesis; in one, the coronary artery, in the other the splenic, was the vessel attacked. In both cases the ulcers had cicatrized, except at the points corresponding to the vessels from which the hæmorrhage proceeded, and to a small extent around these points, Three cases are given in which examination after death showed the existence of cicatrized ulcers in the stomach of persons, who at a former period had suffered from symptoms of gastritis. Such was the case of Professor Belcard. In one case, death was produced by excessive hæmorrhage from the stomach, but on inspection no open vessel was found, and the extreme development of the superficial veins supported the idea that the hæmorrhage had taken place by exhalation.—*Brit. and For. Med. Rev.*, *from Revue Médicale*, *February*, *March*, *and July*, 1838.

---

*Case of Hæmorrhagic Pleurisy. With Remarks.* By Professor Graves.—James Maher, aged twenty-two, admitted September 4th, 1838, in a low and emaciated condition. Has a very troublesome cough, which occurs in paroxysms; sputa scanty and bronchitic; can lie easier on his back than on either side; sweats after sleeping; appetite bad; bowels open; pulse 100, small; respirations hurried.

*Physical Signs.*—On looking at his naked chest, it is evident that the right half of the chest moves much less than the left. Percussion yields a dull sound at the lateral and posterior regions of right side, in which latter region there is bronchial respiration without any rale; in the former there is an absence of respiratory murmur; there is a bronchophony approaching to ægophony posteriorly; whereas, laterally the voice is heard much less than in the natural state; the intercostal spaces are not distended; the left side is normal.

*History.*—States, that about the middle of August he fell in a fit upon his *left* side, and was bled four or five times largely for the apoplectic symptoms. In three days after, he got a severe stitch in his *right* side, for which he was twice copiously bled and blistered, and took some calomel and opium. The symptoms were somewhat abated under this treatment, but the strength of the patient was much reduced.

*September 5th.*

Haustus effervescens ter in die.

6*th.* The patient the same way; there was no rale in the chest this morning when examined.

℞. Pilulæ Hydrarg. gr. iii.
Ext. Opii aquos. gr. ⅓. M.
Fiat pilul. ter in die sumenda.
Vesicatorium magnum parti dolenti.

7th. Was attacked last night with great dyspepsia, cough very bad; spitting up *frothy* serum with a pink tinge; pulse 130, weak; face livid; hands cold; great anxiety; heart beating in a very laboured manner; extensive churning sound heard all over the chest. He was ordered carb. ammoniæ, and shortly after leaving him, raving set in, and death soon followed.

*Autopsy six Hours after Death.*—The right pleural cavity contained about a quart of bloody serum. The posterior portion of the lung was covered with a pretty strong layer of lymph, which was about an eighth of an inch thick, and easily torn off. The same was observed on the parietal pleura opposite to this. The surface of the compressed lung was, as is usual in such cases, wrinkled in many places, a mechanical effect produced by compression. These wrinkles require notice, for in the case before us they imposed on more than one of the spectators, particularly at a part of the posterior surface of the lung, where one of the wrinkles formed, apparently, a deep indenture into the pulmonary substance, *which indenture, containing sero-purulent matter, and covered with a thick layer of lymph, bore a strong resemblance, on a cursory examination, to an abscess.* The bronchial tubes were found to be loaded with a frothy serous fluid, but there was no redness of the bronchial mucous membrane.

The first remark that is suggested by this case, is the tendency which excessive depletion produces to the formation of inflammation. This poor man had been five times bled for a fit of apoplexy, and had been debilitated by various other depletory measures, and in three days afterwards, while lying exhausted and drained of blood, inflammation commences in the pleura, and goes on to a fatal termination unchecked by remedies. Again, another circumstance requires to be noticed, which is, that the nature of the blood and its physical qualities must have been altered by the previous excess of depletion, for we cannot otherwise account for the rather unusual circumstance of the colouring matter being secreted by the inflamed pleura along with the lymph and serum of the blood; in a practical point of view, the sudden occurrence of a churning sound denoting the presence of a serous fluid in the bronchial tubes requires serious attention, for dissection proved that it was not the result of inflammation, but *was produced by a true serous flux into the bronchial tubes*, an event of the most sudden occurrence in the case before us, and which was accompanied by the remarkable rose-coloured serous sputa, which might easily have misled us into the belief that pneumonia existed. Here the colouring matter of the blood presented itself along with the serum, first in the pleural sac, and secondly in the bronchial tubes.—*Dub. Jour. of Med. Science.*

---

*On Enlarged Amygdalæ.* By Prof. Graves.—When common cynanche tonsillaris, scarlatina, measles, or any other disease which induces inflammation of the throat, attacks persons of a scrofulous habit, enlargement of the amygdalæ is a very frequent consequence. In children it is more common than in adults, and when it takes place it requires prompt attention, for if these glands be permitted to become hypertrophied, and to remain so for many years, their size becomes at last considerable, and they may be perceived as large as walnuts, leaving but slight interval between them, so that the disease being confirmed, the patient, when he grows up, is constantly annoyed by an irritation, which, in many, produces a slight hem or occasional hawking, and in all is the source of much inconvenience or even danger, when the person, from cold or any other cause, is attacked with sore throat. Then the inflammation which, under other circumstances, would be moderate, assumes a great degree or violence, the amygdalæ swell suddenly to an excessive size, and the attack is both severe and long continued.

These facts prove the propriety of endeavouring to restrain enlargement of the tonsils in children. After acute diseases, time, with a tonic regimen, country air, with tepid salt-water baths, and sea bathing, will frequently remove this affection, particularly if assisted by gargles, such as warm salt-water, a solution of sulphate of zinc, or infusions of astringent vegetable substances with alum, &c. &c. When these means fail, we may try the daily application of tincture of iodine, mixed with a little treacle.

The principal remedy, however, is the nitrate of silver; many use this in solution, but I prefer Mr. Cusack's method, which is as follows: The solid stick of lunar caustic, or some of the latter in powder, and placed in a proper instrument, must be kept steadily pressed against a particular spot of the enlarged gland; two, three, or five seconds will suffice to secure the formation of a small eschar, which falling out, will leave in the part, when healed, a slight depression like the largest pit formed by a small-pox pustule. When this has been effected, which is usually in about five days, a similar proceeding must take place with the other amygdalæ; and so on with each, turn about, until the desired reduction of size has been accomplished. When the glands are large, this process usually requires about six months; it is slow but sure; and must be intermitted when any accident gives rise to temporary sore throat or to catarrh.

Some use ligatures to reduce these glands in size, and others cut them out; the latter operation is not altogether free from danger, as was proved in the case of a patient of mine, who, contrary to my advice, went to Paris to have it performed. The left amygdala was excised, and the gentleman was very near dying of the consequent bleeding.—*Ibid.*

*Preservation of Bodies for Dissection.*—M. Gannal has recently published a pamphlet on the embalming of bodies, and the preparation of specimens of natural history and morbid anatomy. As M. Gannal has obtained a patent for his method of embalming, we shall say nothing on this part of his work; he has, however, freely communicated the results of his experiments on preservative fluids for the purpose of dissection, of preparations of animals, &c.

After numerous trials with the salts of alum, and various other substances, M. Gannal has selected the sulphate of alumina as being at once the most efficacious and the cheapest materiel that can be employed. Two pounds of the sulphate of alumina dissolved in a quart of water, are sufficient to preserve a dead body in a state of freshness for at least three months. If the weather be very hot it will be necessary to employ the fluid in a greater degree of concentration. The solution of alum is simply injected into the vessels of the subject, and the cost of preservation does not exceed ten pence for each body.

*Lancet, Feb. 2d.*

---

*View of the Operations for Lithotomy, and their Results, in the Hospital of Santa Maria di Loreto in Naples, during a period of sixteen years.* By SALVATORE DE RENZI.

| | SEX. | | AGE. | | | EVENT. | |
|---|---|---|---|---|---|---|---|
| | *Male.* | *Female.* | *Childr.* | *Adults.* | *Aged.* | *Cured.* | *Dead.* |
| 1821 to 1836 | 508 | 15 | 263 | 204 | 56 | 446 | 77 |
| Spring of 1837 | 15 | 0 | 10 | 5 | | 14 | 1 |
| Autumn of 1836 | 15 | 0 | 9 | 6 | | 11 | 4 |
| Total . . | 538 | 15 | 282 | 215 | 56 | 471 | 82 |

This makes the proportion of deaths 1 in 6.7.—*Brit. and For. Med. Rev., from Il Filiatre Sebezio.* Dec., 1837.

---

*Statistical account of the cases of Calculus, treated in the Hospital of St. Mary, St. Petersburg, during 28 years.*—The total number of patients affected with urinary caculus, treated in the hospital, between the years 1808 and 1836, amounted to 1411.

The following table presents a view of the results obtained during the last seven years:—

| Years. | Entries. | Operations. | Cured. | Not cured by Operations. | Died within 1st week. | Died within a Month or more. | Deaths without Operation. |
|---|---|---|---|---|---|---|---|
| 1830 | 63 | 52 | 47 | 9 | 2 | 3 | 2 |
| 1831 | 56 | 52 | 45 | 2 | 4 | 3 | 2 |
| 1832 | 53 | 49 | 43 | 3 | 4 | 2 | 1 |
| 1833 | 86 | 71 | 64 | 11 | 3 | 4 | 4 |
| 1834 | 73 | 69 | 65 | 1 | 2 | 2 | 3 |
| 1835 | 64 | 53 | 46 | 9 | 4 | 3 | 2 |
| 1836 | 74 | 65 | 59 | 6 | 4 | 2 | 3 |
| | 469 | 411 | 369 | 41 | 23 | 19 | 17 |

TABLE *showing the different Ages of the Calculous Patients.*

| Age. | Number of Patients. | Cured. | Not Operated on. | Dead within a Week. | Dead after the lapse of a Month or more of some Acute Disease not connected with Operation. | Deaths without operation |
|---|---|---|---|---|---|---|
| Years. | | | | | | |
| 2 | 28 | 18 | 5 | 1 | 2 | 2 |
| 3 | 51 | 41 | 8 | 1 | 1 | 0 |
| 4 | 54 | 48 | 2 | 1 | 2 | 1 |
| 5 | 38 | 33 | 1 | 2 | 1 | 1 |
| 6 | 39 | 35 | 3 | 1 | 0 | 0 |
| 7 | 38 | 31 | 3 | 2 | 2 | 0 |
| 8 | 35 | 32 | 2 | 1 | 0 | 0 |
| 9 | 22 | 20 | 1 | 0 | 1 | 0 |
| 10 | 21 | 18 | 1 | 1 | 0 | 1 |
| 11 | 13 | 7 | 1 | 3 | 0 | 2 |
| 12 | 18 | 14 | 1 | 2 | 0 | 1 |
| 13 | 10 | 7 | 0 | 3 | 0 | 0 |
| 14 | 15 | 12 | 2 | 0 | 1 | 0 |
| 15 | 10 | 7 | 0 | 0 | 3 | 0 |
| 16 | 3 | 7 | 0 | 0 | 1 | 1 |
| 17 | 10 | 8 | 1 | 0 | 1 | 0 |
| 18 | 9 | 4 | 0 | 1 | 0 | 0 |
| 19 | 4 | 2 | 1 | 0 | 0 | 1 |
| 20 | 8 | 5 | 1 | 1 | 0 | 1 |
| 21 | 3 | 2 | 0 | 1 | 0 | 0 |
| 22 | 6 | 4 | 0 | 1 | 0 | 1 |
| 23 | 7 | 1 | 3 | 0 | 2 | 1 |
| 24 | 2 | 1 | 1 | 0 | 0 | 0 |
| 25 | 2 | 1 | 1 | 0 | 0 | 0 |
| 27 | 4 | 2 | 0 | 0 | 2 | 0 |
| 30 | 1 | 1 | 0 | 0 | 0 | 0 |
| 31 | 1 | 1 | 0 | 0 | 0 | 0 |
| 33 | 2 | 1 | 1 | 0 | 0 | 0 |
| 34 | 2 | 0 | 0 | 1 | 0 | 1 |
| 35 | 2 | 1 | 0 | 0 | 0 | 1 |
| 36 | 1 | 1 | 0 | 0 | 0 | 0 |
| 38 | 1 | 1 | 0 | 0 | 0 | 0 |
| 39 | 1 | 0 | 1 | 0 | 0 | 0 |
| 40 | 2 | 0 | 0 | 0 | 0 | 2 |
| 45 | 1 | 1 | 0 | 0 | 0 | 0 |
| 53 | 1 | 1 | 0 | 0 | 0 | 0 |
| 55 | 1 | 1 | 0 | 0 | 0 | 0 |
| 61 | 1 | 0 | 1 | 0 | 0 | 0 |
| | 469 | 369 | 41 | 23 | 49 | 17 |

The great frequency of calculous affections amongst children in the government of Moskow, depends on the little attention paid them by parents; they stuff the unfortunate infants with potatoes, cakes, and other indigestible stuff; their chambers are small and dirty, and the water in the neighborhood of the villages is not only foul, but contains a quantity of earthy matter. As soon as a child evinces any symptoms of stone they place it under the care of some old female quack, who endeavors to extract the stone by sucking the urethra: this, it is said, frequently succeeds. The rest of their treatment consists in rubbing the abdomen or back with a strong solution of soap, with butter or lard; they shake the child violently, &c.; and it is only when the infant has been exhausted by such barbarous means that he is brought to the hospital.

